# Supplementary material for: SCAPER-Related Autosomal Recessive Retinitis Pigmentosa with Intellectual Disability: Confirming and Extending the Phenotypic Spectrum and Bioinformatics Analyses
Source: Genes (Basel). 2024 Jun 16;15(6):791. doi: 10.3390/genes15060791 (PMC11203295; doi:10.3390/genes15060791)
Supplement: Supplementary file 1 [file genes-15-00791-s001.zip › genes-3047011-supplementary.pdf]

Table S1: Phenotypic and genotypic characteristics of *SCAPER* gene variants of all the published cases including our current cases.

SCAPER variant<br><br>(genotype)	c.352_354	c.2023-2A>G[2]			c.2236dupA	c.1495+1G>A/<br>c.3224delC	c.829C>T/<br>c.3707_3708delCT	c.2377C>T/<br>c.2166-3C>G	c.2179C>T/<br>c.1116delT
SCAPER protein variant	p.(Y118fs*)	NA			p.(I746Nfs*6)	NA/<br>p.(P1075Qfs*11)	p.(R277*)/<br>p.(S1236Yfs*28)	p.(Q793*)/<br>NA	p.R727*/<br>p.V373Sfs*21
Reference	Najmabadi et al., 2011 [2]	Tatour et al., 2017 [5]	Jauregui et al., 2019 [8]	Current cases 2024	Fasham et al., 2019 [9]	Fasham et al., 2019 [9]	Fasham et al., 2019 [9]	Fasham et al., 2019 [9]	Carss et al., 2017 obtained from Fasham 2019 (pt.3) [9]
Ethnicity	Iran	Arab	Arab	Arab	Amish	Cauasian	NA	NA	South Asian
Consanguinity	NA	NA	yes	yes	NA	NA	NA	NA	NA
Number of patients	1	2	1	3	2 (pt.1 +2)	1 (pt.4)	1 (pt.5)	1 (pt.6)	1
**Clinical features:**									
Gender	NA	F,F	M	F,F,F	M,F	F	F	F	F
Age (Years)	NA	24,23	11	30,29,14	13.7, 1.5	31	17	24	28
Height (cm)	NA	NA	NA	147,153,152	166.3, 78.5	NA	NA	162.6	3rd centile
Weight (Kg)	NA	NA	NA	72, 50,86	68.9, 8.6	NA	NA	63.6	25th centile
OFC (cm)	NA	NA	NA	53.5, 53.5, 56	56.4, 47	57	NA	NA	NA
BMI	NA	NA	NA	33.3, 21.4, 37.2	24.9, 14	NA	Obese	24	NA
**Development:**									
Standing (m)	NA	NA	Normal	NA	NA	NA	NA	NA	NA
Waking (m)	NA	normalX2	Normal	15,16,15 (mild delay)	24, 22 (delay)	15	NA	15-18	11 (Noraml)
Speaking (m)	NA	NA	Normal	24,24,18 (delay)	Delay	Delay	NA	Delay	delay
Sitting (m)	NA	NA	Normal	NA	NA	NA	NA	NA	NA
Intellectual disability (IQ)	Yes	mildX2 64, 56	-	52,55, 59 (Yes)	Mod, Mild	Mild	Yes	Mild (50-60)	Yes-Mod
Seizure	NA	NA	-	-	NA	NA	NA	NA	NA
**Behavior Issues:**									
ADHD	NA	+/+	-	+,+,+	+,+	-	NA	+	+
Self-injury	NA	NA	-	-,-,-	NA	-	NA	NA	+
Abnormal neuroimaging (MRI)		Normal/ NA	NA	Nonspecific findings, NA, NA	Normal, NA	NA	Normal	Normal	Normal
Brachydactyly	NA	NA	-	+,+,+	+,+	NA	NA	NA	NA
**EYE:**									
RP (retinitis pigmentosa)	NA	+,+	+	+,+,+	-,-	+	+	+	Yes
Strabismus	NA	NA	NA	+,+,+	NA	NA	NA	NA	NA
Myopia	NA	NA	NA	+,-,-	NA	NA	NA	NA	NA
Vision	NA	RNV	NA	RNV,RNV, RNV	RNV, -	RNV	RNV	RNV	RNV
Cataract	NA	NA	NA	+,-,-	NA	NA	NA	NA	NA
Nystagmus	NA	NA	NA	+,+,-	NA	NA	NA	NA	NA

Glaucoma	NA	NA	NA	+,+,-	NA	NA	NA	NA	NA
Elevator palsy	NA	NA	NA	-,+,-	NA	NA	NA	NA	NA
Facial dysmorphism	NA	-	NA	+,+,+	+,+	-	-	-	-
Skeletal Abnormalities	NA	NA	-	+,+,+ (Short stature)	NA	NA	NA	NA	NA
Speech	NA	NA	Normal	+,+,+ (monotonic nasal)	Delay	Delay	NA	Delay	Delay
Hypotonia	NA	NA	-	+,+,+	NA	NA	NA	NA	NA

SCAPER variant (genotype)	(Chr15: 77,018,886-77,028,490)[1]	c.1096C>T	c.1092dupT	c.1883T>G	c.3781delG c.868_869delGA	c.2806delC	c.2973_2976del	c.1859_1861del c.3656G>A	c.358C>T
SCAPER protein variant	p.(V623fs)	p.(R366*)	p.(V365Cfs*5)	p.(F628C)	p. (V1261Sfs*26)/ p. (E290Sfs*7)	p.(L936*)	p.I991MfsX26	p.E620del p.S1219N	p.(R120*)
Reference	Kahrizi et al., 2019 [7]	Kahrizi et al., 2019 [7]	Kahrizi et al., 2019 [7]	Kahrizi et al., 2019 [7]	Yassin et al., 2024 [10]	Wormser et al., 2019 [6]	Tatour et al., 2017 [5]	Tatour et al., 2017 [5]	Hu et al., 2019 [4]
Ethnicity	Pakistani	NA	NA	NA	Afro-Caribbean	Bedouin	Spanish	Spanish	Baloch
Consanguinity	Yes	yes	yes	NA	No	4 yes, next 4 yes	unrelated	unrelated	yes
Number of patients	2	3	4	1	1	8	1	1	3
**Clinical features:**									
Gender	M,F	F,M,M	M,F,M,M	F	M	F,M,M,M,F,F,F,M	F	M	NA
Age (Years)	18,12	34,32,26	32,12,20,25	7	17	34,28,24,17,48,47,29,10	34	15	NA
Height (cm)	158,150	152,160,172	164,145,163,164	112	NA	145,157,163,155,146,149,132,129	NA	NA	NA
Weight (Kg)	42, 43.5	NA, NA, NA	NA, 40, NA, NA	15	NA	78,78,98,92,87,62,58,30	NA	NA	Normal
OFC (cm)	55, 55	52.5, 51, 55	57.5,55,57.5, 52.5	49	NA	NA	NA	NA	Normal
BMI	16.8 (-2% underweight) , 19.3 (healthy weight)	NA	NA,19 healthy weight, NA, NA	12 (-8% underweight)	NA	37.1, 31.6, 36.9, 38.3, 40.6, 27.9, 33.2, 17.7	NA	NA	NA
**Development:**									
Standing (m)	NA,NA	24,24,20	11,12,11,12	24	NA		NA	NA	NA
Waking (m)	21,21	36,36,36	30,18,12,13	30	NA	NA, NA, NA, NA, NA, NA, NA, NA	24	Delayed	NA
Speaking (m)	24,24	48,48,36	24,18,24,24	36	Delay	NA	NA	NA	NA
Sitting (m)	7,8-9	12,13,NA	8,10,8,9	8	NA	NA	NA	NA	NA
Intellectual disability (IQ)	50,67	31,30,34	40,45,50,40	60	Mild, (88 at age 6 Y)	4 Moderate, 3 Severe, 1Moderate	Moderate	Yes	Yes, all 3
Seizure	-,-	+,+,+	-,-,-,-	-	-	NA	NA	NA	NA
**Behavior Issues:**									
ADHD	-,-	-,-,-	-,-,-,-	-	+	7 NA, last 1+	+	+	NA
Self-injury	+,-	-,-,-	-,-,-,-	-	NA	NA	NA	NA	NA

Others									NA
Abnormal neuroimaging (MRI)	-,+	-,-,-	-,-,-,-	-	NA	7 NP, last 1 abnormal	Normal	NA	NA
Brachydactyly	NA	NA	NA	NA	NA	All 8 +	NA	NA	NA
**EYE:**									NA
RP (retinitis pigmentosa)	+,+	+,+,+	+,+,+,+	+	Mild	7 +, last 1 suspected	+	+	NA
Strabismus	-,-	-,+,-	+,-,+,-	-	-	4 patients +	NA	NA	NA
Myopia	+,+	-,-,-	-,-,-,-	-	mild	4 patients +	NA	NA	NA
Vision	-,-	-,-,-	NB,NB,NB,NA	-	Night problems	Loss of vision 7 and 1 Vision problem	night blindness,	weak.	NA
Cataract	-,-	-,-,-	-,-,-,-	-	-	5 p +	+	-	NA
Nystagmus	-,-	-,-,-	-,-,-,-	-	-	NA	NA	NA	NA
Glaucoma	NA,NA	NA,NA,NA	NA	NA	NA	NA	NA	NA	NA
Elevator palsy	NA	NA	NA	NA	NA	NA	NA	NA	NA
Facial dysmorphism	+,+	+,+,+	-,-,-,-	-	-	- all 8	-	NA	NA
Skeletal Abnormalities	Syndactyly,-	-,-,-	-,-,-,-	-	mild leg length discrepancy	NA	NA	NA	NA
Speech	2y,2y				delay	8 Delay			NA
Hypotonia	+,NA	-,-,-	-,-,-,-	-	NA	NA	NA	NA	NA

Abbreviations: NA: not available; RNV: Reduced night vision

[1]A novel homozygous deletion of around 10 kb on the long arm of chromosome 15 at 15q24.3 (g.77,018,886-77, 028,490, UCSC hg19) which encompassed *SCAPER* exons 15 and 16.

[2]A single base transition positioned in the conserved acceptor splice-site of intron 18.
